# Inclusion Removal Process of Homogeneous CuCr50 Alloy In-Situ Synthesized by Al-Mg Composite Strengthening Reduction Coupling Slagging

**DOI:** 10.3390/ma17143452

**Published:** 2024-07-12

**Authors:** Wang An, Zhihe Dou, Tingan Zhang, Jinru Han

**Affiliations:** 1 Metallurgy School, Northeastern University, Shenyang 110819, China; 2010561@stu.neu.edu.cn (W.A.); xupinhanhao@163.com (J.H.); 2 Key Laboratory of Ecological Metallurgy of Multi-Metal Intergrown Ores of Ministry of Education, Shenyang 110819, China

**Keywords:** CuCr50 alloy, Al-Mg composite reduction, coupled slagging, microstructure, properties

## Abstract

To overcome the problem of Cr_2_O_3_ and Al_2_O_3_ inclusions in CuCr50 alloy prepared by aluminothermic reduction method, in this paper, a novel methodology for strengthening metal–slag separation through in situ slagging is proposed. CuCr50 alloys were prepared by metallothermic reduction using Al and Al-Mg as reducing agents, and the physical properties of the slag, such as viscosity, density, and surface tension, were adjusted by controlling the proportion of CaO in the slagging agent in the raw material to achieve good separation of the slag–metal. The results show that with the ratio of CaO increased, CaO and MgO were coupled to make slag, which combined with Cr_2_O_3_ and Al_2_O_3_ to form CaCr_2_O_4_, MgCr_2_O_4_, and CaAl_4_O_7_ in the slag, thus reducing the content of impurities in the alloy. When RCaO/(CaO + Al_2_O_3_ + MgO) = 20%, the Cr content ranged from 46.61% to 47.09%, the inclusions accounted for 1.60%, the Cr particle size was refined to 20 µm, the number of Cr spherical crystals accounted for 9.88%, the conductivity reached 14.96 MS/m, and the hardness reached 100.23 HB. After heat treatment, the Cr phase was refined in the alloy, the conductivity increased from 14.96 MS/m to 18.27 MS/m, and the hardness increased from 100.23 HB to 103.1 HB. This method is expected to provide an effective method for the preparation of CuCr50 contact materials.

## 1. Introduction

CuCr alloys are the main contact material for medium- and high-voltage and high-current vacuum interrupters due to their excellent characteristics [1,2]. With the development of society, civil and industrial power consumptions are increasing, leading to a tight power supply, and it is expected that the requirements for contact material performance will become more stringent. Furthermore, studies have shown that the size and distribution of the Cr phase in the Cu matrix, the number of impurities, and the density of CuCr alloys have important effects on the material properties [3,4,5]. Therefore, refining the Cr phase grains of CuCr alloys and increasing the alloy density are among the main methods used to improve the properties of contact materials [6].

With the development of contact material preparation technology, three processes for preparing CuCr alloys have been developed: fusion infiltration [7,8,9], powder metallurgy [10,11,12], and arc melting [13]. The fusion infiltration and powder sintering processes disfavor the Cr phase due to mechanical mixing and low-temperature sintering, resulting in CuCr alloys with low density, non-uniform distribution, low hardness, and poor mechanical strength [14,15]. CuCr alloys prepared by arc melting have the characteristics of high density and low impurity content, but the cost of alloy production is high due to the large investment in equipment and the raw materials used: pure Cu powder and pure Cr powder [16,17]. To make a breakthrough in the preparation of high-performance CuCr alloys, different new processes have been developed by domestic and foreign scholars. Feng et al. [18] used pure Cu powder (<51 µm) and pure Cr powder (<74 µm) as raw materials and ball-milled them in a Simoloyer01-2L horizontal high-energy ball mill protected by Ar gas for 4~6 h. Then, the material was hot pressed in vacuum hot-pressing equipment at a temperature of 500 °C and a pressure of 800 MPa, with a holding time of 1 h~1.5 h. The sintered material was then subjected to vacuum hot-pressing equipment at a pressure of 800 MPa. Nanocrystalline CuCr25 and CuCr50 alloys were successfully prepared, the vacuum arc cut-off value of CuCr alloys was 17–35% of that of conventional alloys, and the life of the vacuum arc increased accordingly. Lahiri, Indranil et al. [19] successfully prepared high-strength and high-density CuCr10 and CuCr50 alloys by ball milling for 150 h on a FritschPulversette P5 planetary ball mill followed by uniaxial compaction and explosive sintering. From the perspective of developing key technologies for the low-cost, short-process preparation of non-segregated CuCr alloys, Zhang et al. [20] proposed the process of CuCr alloy preparation by aluminum thermal reduction-electromagnetic melt casting of large homogeneous CuCr alloys. This process was successful in the preparation of large CuCr alloy ingots without segregation, but there was porosity and inclusion defects in the alloy. Zhang et al. further proposed subjecting self-propagating-obtained CuCr alloy ingots to vacuum self-consumption remelting refining to eliminate defects, but the defects were not effectively removed. To completely eliminate the defects present in CuCr alloys, the mutually soluble CuCr alloy melt obtained by aluminum thermal reduction was subjected to magneto-electric coupling refining to fully float the pores and inclusions in the alloy, and rapid cooling of the water-gas composite was used to suppress segregation; finally, dense and homogeneous CuCr alloy ingots were obtained. To simplify the process and shorten the preparation time, the porosity problem in the preparation of CuCr50 alloys by aluminum thermal reduction method must be addressed. Han et al. [21] successfully solved the problem of porosity defects by adding excess Cr_2_O_3_to the raw material for coupled slagging in the preparation of CuCr50 alloy by aluminum thermal reduction method. This led to a large number of inclusions in the alloy containing Cr_2_O_3_ due to the addition of excess Cr_2_O_3_.

The factors affecting slag–metal separation were analyzed according to physical properties, such as the viscosity and density of the MgO-CaO-Al_2_O_3_ ternary slag system investigated by Shi [22]. The viscosity, density, and solidification temperature of the slag were the lowest when *w*(CaO):*w*(Al_2_O_3_) = 1:2 and *w*(MgO) = 3%, and the slag–metal separation was the best at this time. To overcome the problem of Cr_2_O_3_ inclusion removal during the preparation of CuCr50 alloys by aluminum thermal method, in this paper, we use Al-Mg composite reducing agent to strengthen the reduction coupling slagging process, focusing on the design of the slag system during the reduction melting process and its influence on the uniformity and properties of the alloy to achieve a breakthrough in the theoretical method of source regulation of one-step in situ reduction synthesis of homogeneous CuCr50 alloys. It provides technical and theoretical support for the preparation of high performance CuCr50 alloy by the metal thermal reduction method, and a CuCr50 contact material with better performance than GB/T26867-2011 [23] is prepared.

## 2. Experimental

### 2.1. Materials

In the experiment, CuO (purity ≥ 99.7%, granularity: 25 μm) and Cr_2_O_3_ (purity ≥ 99.7%, granularity: 24.5 μm) were used as raw materials, KClO_3_ (purity ≥ 99.6%, granularity: 85 μm) was used as a heating agent, CaO (purity ≥ 98.0%) was used as a slagging agent, and Mg (purity ≥ 99.0%, granularity: 85 μm) was used as an ignition agent. The reducing agent was a mixture of 87.5% Al powder (purity ≥ 99.5%, granularity: 2.5 mm) and 12.5% Al-40Mg alloy powder (purity ≥ 99.7%, granularity: 0.18–0.84 mm), with the aim of obtaining an Al-5Mg alloy powder reducing agent and thus adding MgO in situ.

### 2.2. Experimental Procedure

**Figure 1:**
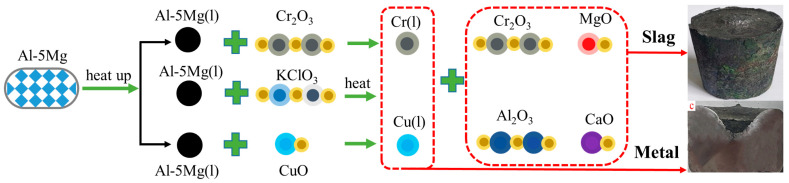
Schematic diagram of the process for preparing CuCr50 alloy by Al-Mg composite reduction.

The experimental materials CuO, Cr_2_O_3_, KClO_3_, and CaO were dried in a Nabertherm oven (TR 1050) imported from Germany at 423 K for 24 h. All the experimental ingredients were mixed proportionally (Table 1 Different CaO additions) and then preheated at 373 K for 1 h. The mixture was filled into a graphite funnel, and 3–5 g of Mg powder was sprinkled on the surface of the mixture, which was ignited by an open flame to initiate the spontaneous combustion reaction. When the first batch of material reaction was completed and the material flowed into the graphite casting mold with a crystallizer, the remaining material was continuously added to the graphite funnel, and the residual temperature was used to trigger a self-propagating reaction to obtain the final mutual solubility of the high temperature CuCr50 alloy melt. The rotating magnetic field on the outside of the crystallizer was turned on, the magnetic field rotation frequency was 18Hz, the stirring time was 10 min, the sample was subjected to water cooling and rapid solidification to obtain a Φ60 mm×h45 mm CuCr50 alloy ingot, and the sample was cooled to room temperature. Finally, the reduction slag and alloy ingots were collected for testing and analysis. Figure 1 is schematic diagram of the process for preparing CuCr50 alloy by Al-Mg composite reduction.

Then, the prepared alloy ingots were subjected to solid solution treatment at 975 °C for 1 h under argon atmosphere, followed by quenching in water. Finally, aging treatment was carried out in a tube furnace under argon atmosphere for 2 h at a temperature of 500 °C [24].

### 2.3. Analysis and Characterization

The chemical analysis method (GB/T 1467-2008) [25] was used to analyze the contents of different parts of the alloy. According to the GB/T1423-1996 [26] alloy density test method, the alloy density was measured. The physical phase of the reduced slag was analyzed by XRD with a Bruker instrument (Germany Bruker). The content of each oxide in the reduced slag was analyzed using X-ray fluorescence (XRF) on a model ZSX Primus IV instrument manufactured by Rigaku, Japan. The microstructure was characterized by ZEISS Axio Imager M2m and SEM/EDS (MIRA3 Tescan). The alloy conductivity and hardness were measured by a model FD-102 eddy current conductivity meter (YS/T 478-2005) [27] and HBS-3000Z Bush hardness tester (GB/T 231.1-2018) [28], respectively.

## 3. Results and Discussion

### 3.1. Theoretical Calculations

#### 3.1.1. Calculation of the Reaction Process

**Figure 2:**
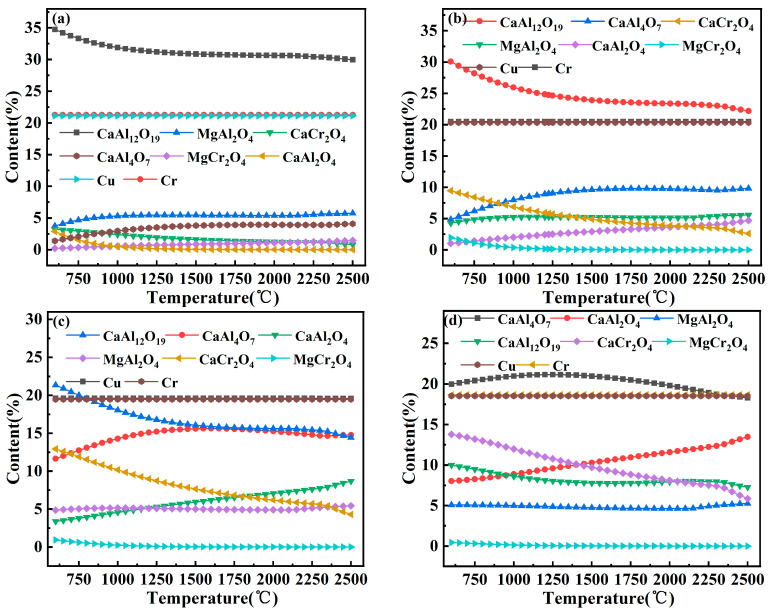
Theoretical reduction products of different ingredient ratio systems: (**a**) R = 10%, (**b**) R = 15%, (**c**) R = 20%, (**d**) R = 25%.

The Equilibrium Compositions module of the HSC 6.0 thermodynamic software was used to calculate the phase equilibrium for different CaO ratios (RCaO/(CaO + Al_2_O_3_ + MgO)), as shown in Figure 2. The reduced metal product had a 1:1 mass ratio of Cu to Cr. When the CaO content of the raw material was increased to 20% and 25%, the slag had more CA, CA2, and CaCr_2_O_4_ low-melting-point phases, and the slag had good fluidity, which facilitates the effective separation of clean metal from the slag by the alloy.

#### 3.1.2. Precipitation Process of Reduced Slag

The actual composition of the reduction slag with different ratios was analyzed using XRF, as shown in Table 2. The melting temperatures of different reduction slags were calculated with the help of the Phase Diagram module in the FactSage 8.1 software and the chosen database: FToxid. With the increase in the CaO percentage in the reduction slag, the slag phase changes from the high-melting-point CaAl_12_O_19_ phase to the low-melting-point CaAl_4_O_7_ phase, and the melting temperature of the slag decreases (Table 3).

**Figure 3:**
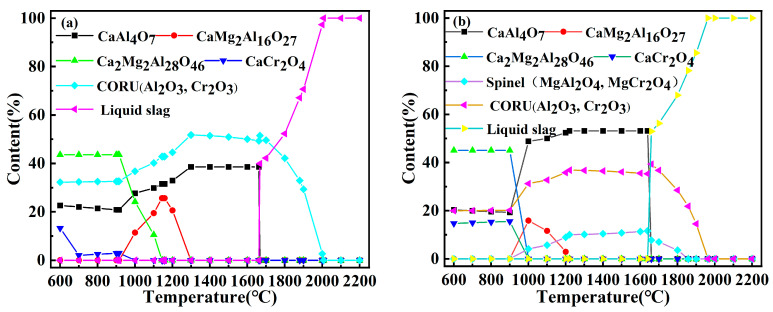

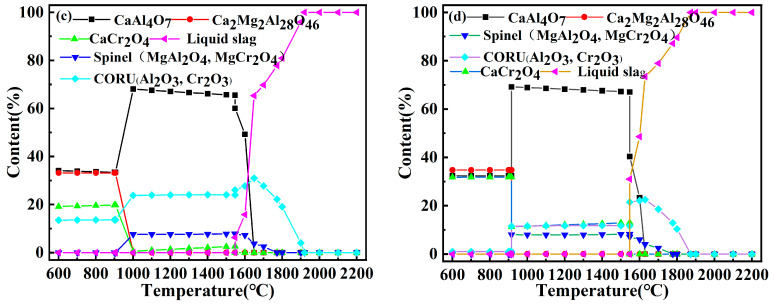
Phase transition of slag with different CaO additions: (**a**) R = 10%, (**b**) R = 15%, (**c**) R = 20%, (**d**) R = 25%.

**Figure 4:**
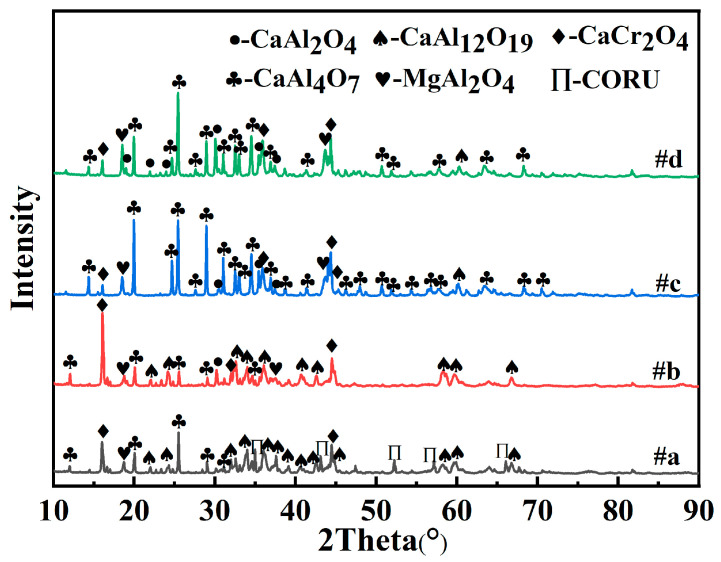
X-ray diffraction of patterns reduced slags with different CaO ratios: (**a**) R = 10%, (**b**) R = 15%, (**c**) R = 20%, (**d**) R = 25%.

The phase transformation patterns of the slag during the slow cooling off process were calculated, as shown in Figure 3. With decreasing temperature, the high-melting-point CORU, the composite phase of Al_2_O_3_ and CaO, precipitates in the slag first, and then the calcium aluminate phase and the MgAl_2_O_4_ and MgCr_2_O_4_ spinel phases began to precipitate, and the liquid slag decreased. When the temperature was reduced to 1200 °C, CaAl_12_O_19_ combined with MgAl_2_O_4_ to form Ca_2_Mg_2_Al_28_O_46_ (2CA6-2MA) and CaMg_2_Al_16_O_27_ (CA6-2MA). When the temperature continued to decrease to 900 °C, the spinel phase disappeared in the reduced slag, and Cr_2_O_3_ combined with CaO to form CaCr_2_O_4_. With increasing CaO, the CORU phase content decreased, CaCr_2_O_4_ increased, and the CORU phase disappeared at R = 25%; thus, the CaCr_2_O_4_ phase was more likely to exist stably. The reduced slag transformation process is consistent with the XRD results (Figure 4).

#### 3.1.3. Calculation of the Reduction Slag Physical Properties

The physical properties of reducing slag have a great influence on the separation effect of slag–metal. Therefore, the lower the density of the slag, the greater the density difference between the slag and the metal, the greater the surface tension, and the easier the settlement and separation. The liquid phase line of CuCr alloy is around 1750 °C, and if the temperature below the liquid phase line is not completely separated, there will be impurities in the metal; so, the theoretical density of the reduced slag from 1750 °C to the complete melting temperature was calculated.

According to the classical assumptions of slag coexistence theory [29], a thermodynamic model was developed, and the surface tension of the CaO-Al_2_O_3_-Cr_2_O_3_-MgO slag system was calculated based on the Bulter equation:

(1) $$\sigma =\sigma_{i}^{Pure}+\frac{RT}{A_{i}}\ln\frac{N_{i}^{Surf}}{N_{i}^{Bulk}}$$

where *σ*—the surface tension of the slag system; *σ_i_*—the surface tension of the pure substance i surface tension; *N_i_^Surf^* and *N_i_^Bulk^*—the mass action concentrations of *i* in the surface phase and the bulk phase, respectively; *A_i_* is the molar surface area of *i*.

Table 4 is expressions of structural components and action concentration in CaO-Al_2_O_3_-Cr_2_O_3_-MgO slag system based on coexistence theory. The composition of CaO-Al_2_O_3_-Cr_2_O_3_-MgO slag is defined as follows: *b*_1_ = *Σn*_CaO_, *b*_2_ = *Σn*_Al2O3_, *b*_3_ = *Σn*_Cr2O3_, and *b*_4_ = *Σn*_MgO_. The total equilibrium mole number of all structural components in CaO-Al_2_O_3_-Cr_2_O_3_-MgO can be expressed as follows:

(2) $$\sum n_{i}=2n_{1}+n_{2}+5n_{3}+2n_{4}+n_{5}+n_{6}+n_{7}+n_{8}+n_{9}+n_{10}+n_{11}+n_{12}+n_{13}(mol)$$

The mass balance equation is as follows:

(3) $$b_{1}=(0.5\times N_{1}+3\times N_{5}+N_{6}+N_{7}+N_{8}+N_{9}+2\times N_{12}+3\times N_{13}+N_{14})\times \sum n_{i}$$

(4) $$b_{2}=(N_{2}+N_{5}+6\times N_{6}+N_{7}+2\times N_{8}+N_{10}+14\times N_{12}+2\times N_{13}+8\times N_{14})\times \sum n_{i}$$

(5) $$b_{3}=(0.2\times N_{3}+N_{9}+N_{11})\times \sum n_{i}$$

(6) $$b_{4}=(0.5\times N_{4}+N_{10}+N_{11}+2\times N_{12}+N_{13}+2\times N_{14})\times \sum n_{i}$$

(7) $$N_{1}+N_{2}+N_{3}+N_{4}+N_{5}+N_{6}+N_{7}+N_{8}+N_{9}+N_{10}+N_{11}+N_{12}+N_{13}+N_{14}=1$$

**Figure 5:**
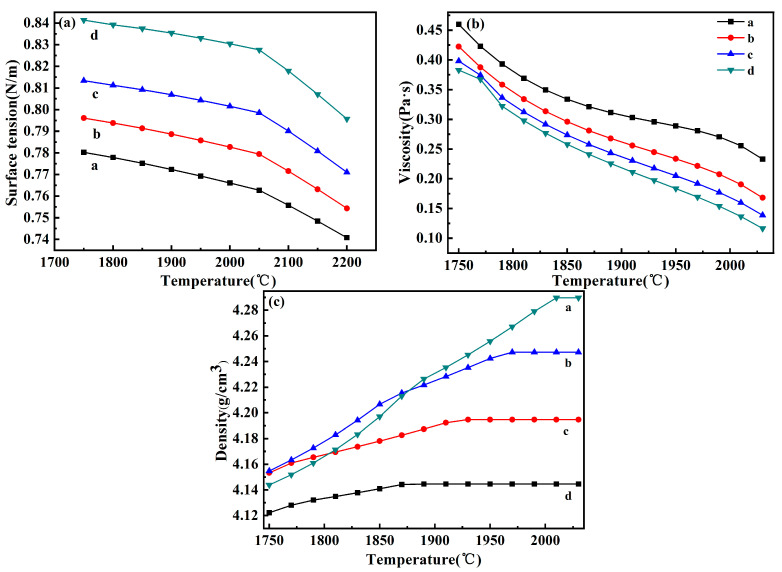
Physical properties of reduced slag with different CaO additions ((**a**)—surface tension, (**b**)—viscosity, (**c**)—density).

Figure 5. Physical properties of reduced slag with different CaO additions. With in-creasing CaO, the surface tension of the reduced slag increases. The in situ reduction of Al-Mg composites to MgO and the addition of CaO led to an increase in O^2−^ in the slag. The chemical reaction between Al_2_O_3_ and O^2−^ can be carried out more easily, which promotes the [AlO_4_]^−^ tetrahedral depolymerization of the mesh structure. The radius of the complex ions decreases, thus increasing the surface tension of the reduced slag. With increasing temperature, the interionic force increased, and the surface tension of the slag decreased. When the reduction slag is completely melted, the surface tension of the 799 mN/m Al-Mg composite reduction slag is greater than that of the 766 mN/m aluminum thermal reduc-tion slag, which facilitates slag–metal separation.

The Iida viscosity prediction model was used to calculate the variation in viscosity for different reduction slags [30], as shown in Figure 5b.

(8) $$\mu =A\mu_{0}\exp\left(\frac{E}{B_{i}}\right)$$

(9) $$\mu_{0}=\sum \mu_{0i}x_{i}$$

(10) $$B_{i}=\frac{\sum {\left(a_{i}w_{i}\right)}_{basic-oxides}}{a_{Al_{2}O_{3}}w_{Al_{2}O_{3}}}$$

where *μ*_0_—viscosity; *A*, *E*—temperature function; *μ_0_*—ideal viscosity when the components do not affect each other; *T*—absolute temperature; *μ*_0*i*_—mole fraction of the component; *x_i_*—enthalpy of melting of the component; *a_i_*—parameters required for the computation of *B_i_*.

The viscosity decreases with increasing temperature, and there is no obvious turning point in the viscosity–temperature curve showing long-slag characteristics, which provides a better drive for slag–metal separation. As the proportion of CaO increases, CaO combines with Cr_2_O_3_ and Al_2_O_3_ to form CaCr_2_O_4_ and CaAl_4_O_7_, respectively, and in situ added MgO combines with Cr_2_O_3_ to form MgCr_2_O_4_, which breaks down the high-melting-point CORU phase, and the slag viscosity decreases. When the reducing slag is completely melted, the viscosity of the Al-Mg composite reducing slag is lower than that of the aluminum thermally reducing slag by 0.37 Pa·s [4], which facilitates the separation of slag–metal.

The theoretical density of the slag was calculated from the liquid-phase line of the CuCr alloy between 1750 °C and the complete melting temperature according to the theoretical estimation formula of the density of the slag [31].

(11) $$\rho_{slag}=\sum X_{i}\rho_{i}$$

where *ρ_slag_*—the density of slag, g/cm^3^; *X_i_*—the mass percentage of slag element *i,* %; *ρ_i_*—the density of slag element *i*, g/cm^3^.

With increasing temperature, the density of the reduced slag increased. As the temperature increased, the CORU phase, MgAl_2_O_4_, and MgCr_2_O_4_ phases began to melt, resulting in an increase in the number of oxides in the molten state in the slag and an increase in density. The full melting densities of reduced slags a, b, c, and d were 4.29 g/cm^3^, 4.24 g/cm^3^, 4.19 g/cm^3^, and 4.15 g/cm^3^, respectively. When the amount of CaO in the raw material was greater than 15%, the density of molten slag was less than that of aluminum thermally reduced slag (4.28 g/cm^3^) [4], and the density of slag was much less than that of molten metal (8.06 g/cm^3^). Therefore, it is easy to realize slag–metal separation.

### 3.2. Reduction Slag Phase Transition

**Figure 6:**
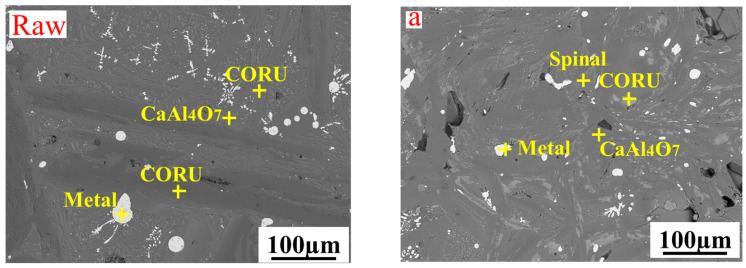

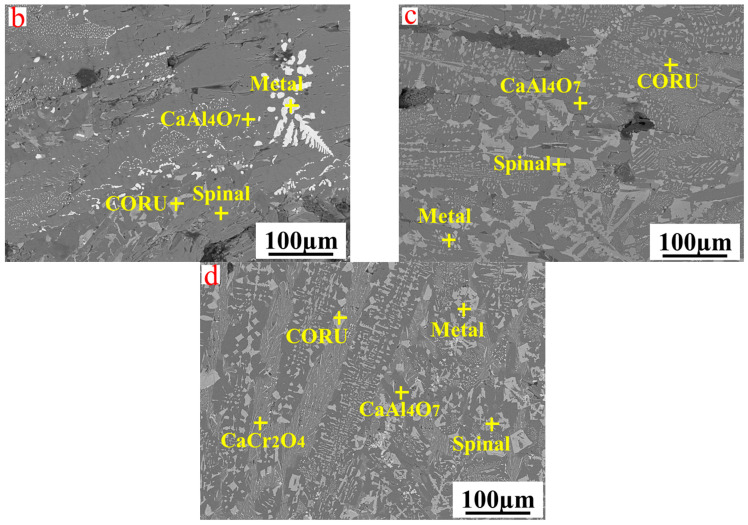
Microscopic morphology of reduced slag with different CaO ratios (**raw**): aluminum thermal reduction slag: (**a**) R = 10%, (**b**) R = 15%, (**c**) R = 20%, (**d**) R = 25%.

Figure 6 shows the microscopic morphology of the different slag reduction ratios after slow cooling. The amount of metal inclusions in the aluminum thermal reduction slag was greater than that in the Al-Mg composite reduction slag system, and the main phase in the reduction slag CORU high-melting-point phase was not conducive to slag metal separation. In the Al-Mg composite reduction slag system, with increasing CaO, the amount of metal particles in the slag decreased, the alloy yield increased, and the separation effect of the slag–metal mixture clearly improved. At low CaO ratios, the slag phase crystallization is not obvious, and the main phases are matrix CaAl_4_O_7_, irregular spinel (MgAl_2_O_4_, MgCr_2_O_4_), and CORU (Al_2_O_3_, Cr_2_O_3_). When the CaO ratio increased to 20%, the slag-phase crystallization improved significantly, the shape of the phase became more regular and clearer, the spinel was a fish bone, and the CORU was rhombic and slate-like. With CaO increasing to 25%, a large amount of needle-like CaCr_2_O_4_ was present in the structure, and the spinel and CORU phases were uniformly and finely distributed. This was mainly due to the increase in CaO reduction and slag melting point, and the slag from the reaction system temperature (2848 K) down to the melting point time was extended, giving the slag phase growth time crystallinity, and high melting point slag solidification was faster, similar to water-quenched slag crystallinity being low.

**Figure 7:**
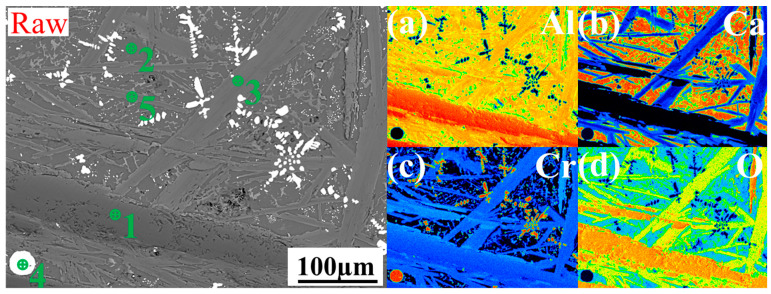

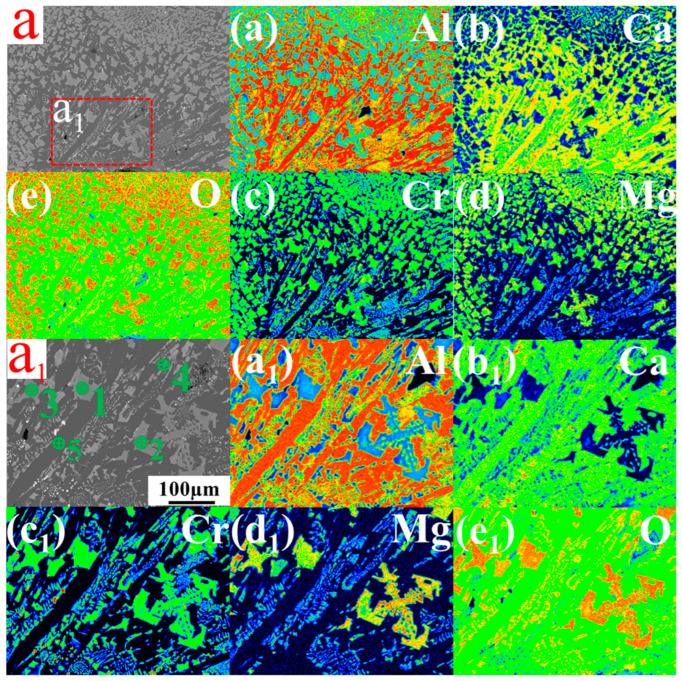
EPMA of mapping for the slag–metal interface (**raw**): aluminum thermal reduction slag, (**a**)-Al, (**b**)-Ca, (**c**)-Cr, (**d**)-O element; (**a**): Al-Mg composite reduction slag, (**a**)-Al, (**b**)-Ca, (**c**)-Cr, (**d**)-O, (**e**)-Mg element; (**a_1_**): a selection enlargement, (**a_1_**)-Al, (**b_1_**)-Ca, (**c_1_**)-Cr, (**d_1_**)-O, (**e_1_**)-Mg element.

Figure 7 shows the EPMA of aluminum thermal reduction slag and composite reduction slag. The aluminum thermal reduction slag mainly consisted of matrix CaAl_4_O_7_ (point 2) and long strips of Al_2_O_3_ and Cr_2_O_3_ composite phases (points 1, 3, 5), with a large amount of metal Cr inclusions in the slag (point 4). The Al-Mg composite reduction slag matrix was CaAl_4_O_7_ (point 5), most of the physical phases were MgAl_2_O_4_ and MgCr_2_O_4_ composite phases (points 1, 2, 3), as well as a trace amount of the Al_2_O_3_ and Cr_2_O_3_ composite phase (point 4), and no obvious metallic Cr inclusions were found in the slag. This is mainly due to the in situ addition of MgO, which transforms the high melting point CORU phase into the spinal phase. The viscosity and density of the reduced generated slag decreases, and surface tension increases, increasing the slag–metal separation.

### 3.3. Analysis of the CuCr50 Alloy

#### 3.3.1. Uniformity Analysis of the CuCr50 Alloy Ingot

**Figure 8:**
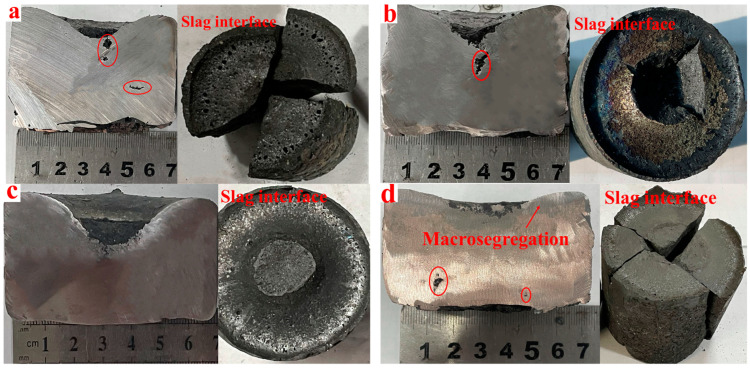
Macroscopic images of the interface and alloy of CuCr alloy slag–metal synthesized with different CaO ratios: (**a**) R = 10%, (**b**) R = 15%, (**c**) R = 20%, (**d**) R = 25%.

**Figure 9:**
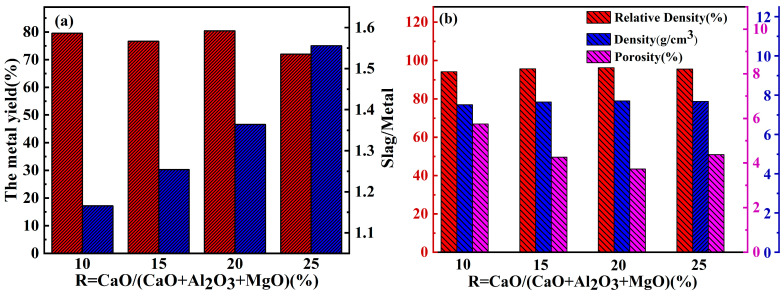
Yield and densification of CuCr50 alloys with different ingredients: (**a**) metal yield, (**b**) alloy density.

Figure 8 shows the slag–metal interface of the CuCr50 alloy synthesized in situ under 15% CaO and 20% CaO. With increasing CaO, the smoothness of the slag–metal surface increased, the slag entrapment at the metal interface decreased, the internal pores of the alloy decreased, and the slag–metal separation was completed. According to the GB/T1423-1996 [25] alloy density test method, the alloy density was measured, as shown in Figure 9b, and the density of the alloy was highest at 20%. As the amount of CaO incorporated in the raw materials increased, the viscosity and density of the reduced generated slag decreased, and the surface tension increased, which is characteristic of long slag and thus easy slag–metal separation. When the ratio of CaO increased to 25%, the fluctuation of Cu and Cr mass fractions in the upper and lower parts of the alloy increased (Table 5). Due to the excessive addition of CaO generating too much slag (Figure 9a), the reduction slag had a low thermal conductivity, resulting in a small degree of subcooling of the alloy melt solidification becoming slower, giving time for the Cr to fully float, and causing the alloy to appear to be macroscopically segregated.

**Figure 10:**
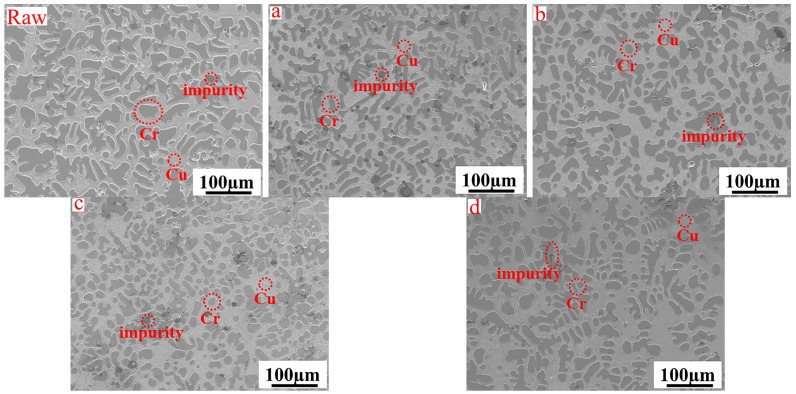
Microstructure of CuCr alloys synthesized with different CaO ratios (**raw**): aluminum thermal reduction synthesis of CuCr50 alloy: (**a**) R = 10%, (**b**) R = 15%, (**c**) R = 20%, (**d**) R = 25%.

**Figure 11:**
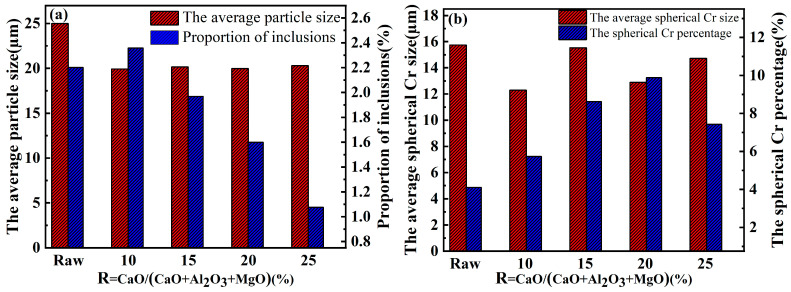
Proportion of inclusions and average particle size of the Cr phase in CuCr alloys synthesized with different CaO ratios (raw: aluminum thermal reduction alloy; (**a**) average Cr phase particle size and number of inclusions, (**b**) number of Cr spherical crystals).

Figure 10 shows the SEM images of the in situ synthesized CuCr alloys with different CaO ratios, and the statistics of the number of inclusions and the sizes of the Cr phases in the alloys obtained by Image Pro Plus (IPP) are shown in Figure 11. The Cr phase grains of the CuCr50 alloy synthesized by Al-Mg composite reduction are smaller than those synthesized by Al thermal reduction (25 µm–20 µm). The number of Cr phase spherical crystals was increased (4.11–9.88%), the average grain size of spherical crystals was decreased (15.74 µm–12.89 µm), the oxygen content was decreased (0.755–0.579%), and an increase in the number of spherical crystals could increase the hardness of the alloy and play a role in diffusion strengthening. As the amount of CaO increases, the number of inclusions in the metal decreases (2.36–1.08%) in the metal. This is mainly because the increase in CaO combined with Al_2_O_3_ and unreduced Cr_2_O_3_, CaAl_4_O_7_, and CaCr_2_O_4_ are generated so that the melting point and viscosity of the reduced slag are reduced and the surface tension is increased; thus, the metal–slag separation is better.

**Figure 12:**
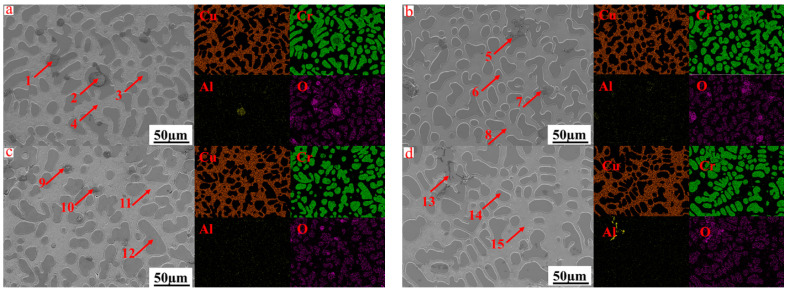
Surface scanning distribution of CuCr alloys synthesized with different CaO ratios: (**a**) R = 10%, (**b**) R = 15%, (**c**) R = 20%, (**d**) R = 25%.

**Figure 13:**
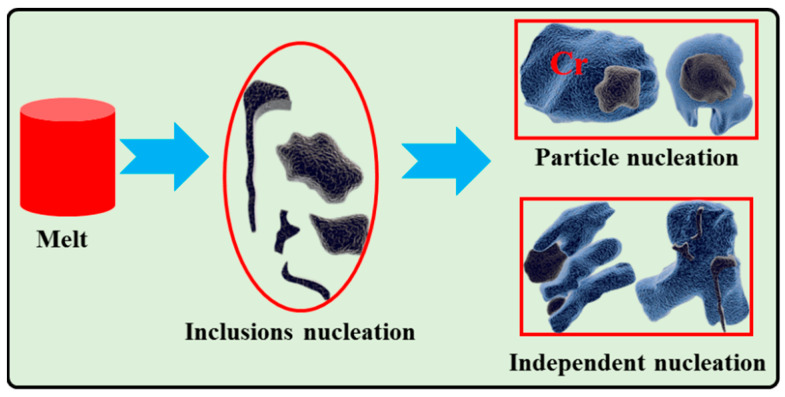
Schematic diagram of the nucleation–growth mechanism of inclusions and Cr phases.

Figure 12 shows the mapping of the CuCr alloys synthesized in situ with different CaO ratios, and Table 6 shows the mapping results. The inclusions at 10%, 15%, and 20% CaO are mainly Cr_2_O_3_ inclusions containing a small amount of Al_2_O_3_. The distribution of inclusions is divided into two forms: one is concentrated in the middle of the Cr particles, providing mass points for Cr particle nucleation; the other is distributed at the grain boundaries between the Cr phase and the Cu matrix. For the first reason, the inclusions melting point is higher than the Cr phase, the inclusions first nucleation growth, and then the Cr phase inclusions as particle nucleation. The second formation mainly occurs because the CuCr alloy is a “pseudo-alloy”, and the two are not compatible with each other: Cr melts and a small number of inclusions are combined with growth, and the Cu melts are wrapped together (Figure 13). Inclusions exist between the interfaces of the two phases. When the CaO content increases to 25%, a small amount of needle-like Al_2_O_3_ inclusions are distributed in the Cr particles. According to the X-ray diffraction pattern of the slag CaCr_2_O_4_ peak enhancement, the CaO and Cr_2_O_3_ in the inclusions form the CaCr_2_O_4_ phase in the reduction slag, and the inclusions are decreased in the CuCr50 alloy.

#### 3.3.2. Crystal Structure and Properties Analysis of CuCr50 Alloy

**Figure 14:**
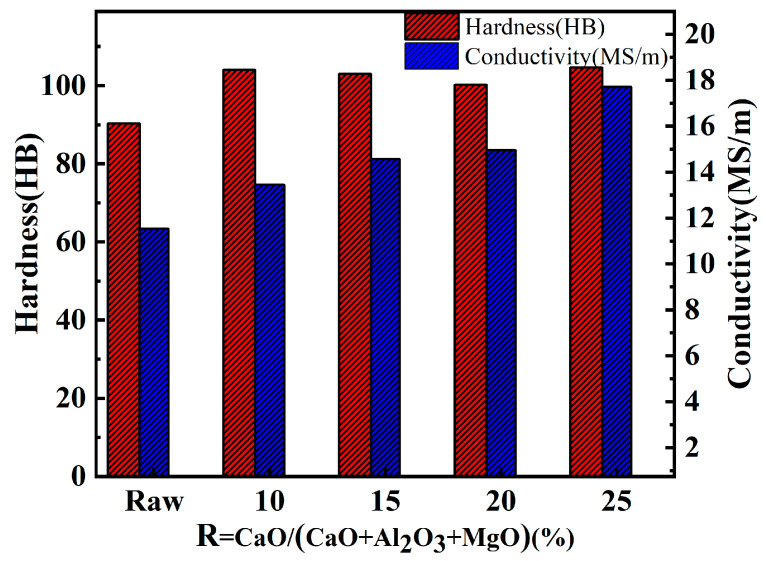
Conductivity and hardness of CuCr alloys synthesized in situ with different CaO ratios (raw: aluminum thermal reduction synthesis of CuCr50 alloy).

The conductivity and hardness of the in situ synthesized CuCr50 alloy were tested for different raw material ratios (Figure 14). The hardness of the alloy is approximately 100 HB, which is higher than that of GB/T26867-2011 [23]. Compared with the synthesis of CuCr50 alloys by aluminum thermal reduction, the hardness of the alloy increased. According to the Orowan strengthening equation [32,33], there was a decrease in the Cr phase grain size from 25 µm to 20 µm, there was an increase in the number of Cr phase spherical crystals from 4.11% to 9.88%, and there was a decrease in the average grain size of the spherical crystals from 15.74 µm to 12.89 µm. The size of Cr particles decreases, which plays a role of dispersion strengthening, so that the hardness of the alloy increases. 

With increasing CaO, the conductivity of the alloy shows an increasing trend, and it is lower than that of GB/T26867-2011 [23] at 10%, 15%, and 20%. With the large cooling rate of alloys synthesized by the thermal reduction of metals, the concentration of Cr atoms solidly dissolved in the Cu matrix is high, with a solid solubility of 2.1%. (Table 7). The dissolution of Cr atoms causes lattice distortion in Cu, increasing the scattering effect of the lattice on electrons and leading to a decrease in conductivity [34,35,36]. With increasing CaO content, the number of inclusions in the alloy decreases (2.36–1.08%), the porosity decreases (5.75–3.73%), and the alloy conductivity increases (13.45 MS/m–14.96 MS/m).

The conductivity and hardness of the CuCr50 alloy synthesized using Al-Mg composite reducing agent were better than those of the CuCr50 alloy synthesized by aluminum thermal reduction. As the hardness increased from 90.3 HB to 100.23 HB, the conductivity increased from 11.54 MS/m to 14.96 MS/m. This is mainly because the Al-Mg composite reduces the in situ addition of trace amounts of MgO and decreases the melting point of the reduction slag (2026.99 °C–1919.53 °C), so the density and viscosity of the reduced slag decrease and the surface tension increases, enhancing the slag–metal separation effect. At the same time, the Cr phase particle size can be refined, and the number of spherical crystals increases to strengthen the dispersion.

**Figure 15:**
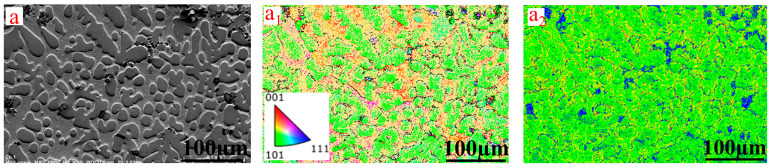

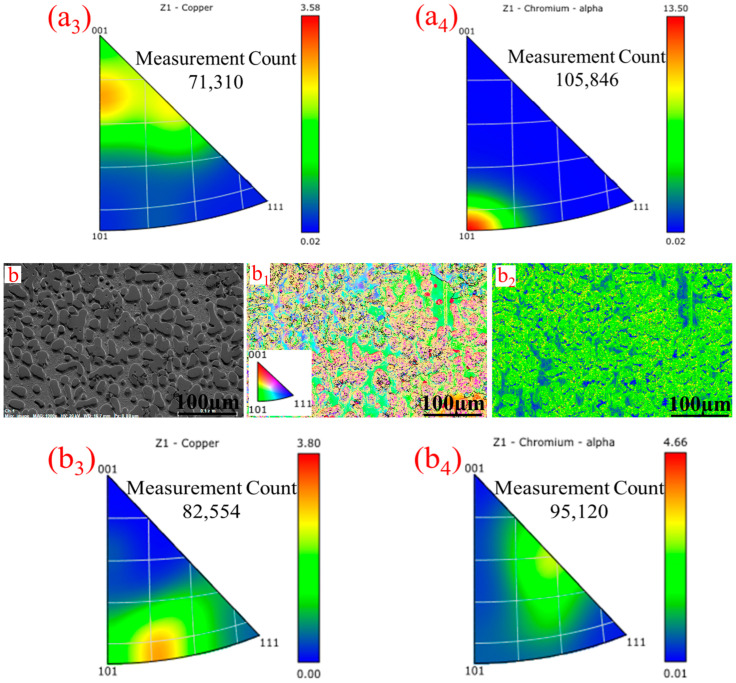
In situ synthesis of CuCr50 alloy EBSD by Al-Mg composite reduction at 20% CaO ratio (**a**) before heat treatment, (**a_1_**) EBSD, (**a_2_**) KAM, (**a_3_**) Cu inverse pole figure, (**a_4_**) Cr inverse pole figure; (**b**) after heat treatment, (**b_1_**) EBSD, (**b_2_**) KAM, (**b_3_**) Cu inverse pole figure, (**b_4_**) Cr inverse pole figure.

Figure 15 shows the EBSD of CuCr50 alloys synthesized in situ by Al-Mg composite reduction before and after heat treatment at a 20% CaO ratio and heat treatment at 975 °C for 1 h + 500 °C for 2 h [24]. The alloy contains bcc Cr and fcc Cu, and no obvious solid solution. The orientation of the Cu and Cr phases before heat treatment is single, dominated by the Cr[101] direction, and the Cu[001] direction, with 2–10° grain boundaries accounting for 91.8% and 10–60° grain boundaries accounting for 8.21% of the small-angle grain boundaries. Due to the faster cooling of the CuCr alloy melt, there are a large number of dislocations in the cast state. After heat treatment, the Cr phase grains are obviously refined, and a large number of spherical Cr particles appear in the Cu matrix. Meanwhile, the Cu, Cr two-phase orientation transforms, the Cu phase changes from the original [001] to [101] direction, and the Cr phase changes from [101] to [001] and [111] directions. With 2–10° grain boundaries accounting for 88.1%, 10–60° grain boundaries accounting for 11.9% of the large-angle grain boundaries and accounted for more than an obvious increase, and the number of dislocations in the Cu matrix decreased. After heat treatment, a large number of Cr particles precipitated from the Cu matrix, which caused the Cu lattice distortion to disappear [37,38], and conductivity increased from 14.96 MS/m to 18.27 MS/m. After heat treatment, the Cr particle size decreased (15.1 μm–13.5 μm) and the number of spherical Cr grains increased, with a uniformly dispersed distribution in the Cu matrix, thus increasing the strength of the alloy from 100.23 HB to 103.1 HB.

## 4. Conclusions

In this paper, CuCr50 alloys were synthesized in situ using Al-Mg composite reducing agent coupled with slagging, and the effects of CaO addition and in situ generation of MgO on the physical properties of the reduced slag and the homogeneity of the microstructure were investigated. The main conclusions are as follows:

1. Compared with aluminum thermal reduction slag, the surface tension of Al-Mg composite reduction slag is greater, and the viscosity and density are lower and more conducive to the separation of gold slag.
2. When the CaO ratio was increased to 25%, a large number of needle-like CaCr_2_O_4_ phases appeared, CaO and Cr_2_O_3_ combined to form CaCr_2_O_4_, and in situ generated MgO combined with excess Cr_2_O_3_ to form MgCr_2_O_4_ in the slag. In the alloy, the inclusions were transformed into a small amount of needle-like Al_2_O_3_ inclusions, which improved the cleanliness of the alloy.
3. Compared with the CuCr50 alloy synthesized by aluminum thermal reduction, the size of the Cr phase decreased to 25 µm–20 µm, the number of Cr phase spherical crystals increased (4.11–9.88%), the average grain size of spherical crystals decreased (15.74 µm–12.89 µm), the number of inclusions decreased (2.36–1.08%), and the oxygen content decreased (0.755–0.579%).
4. When RCaO/(CaO + Al_2_O_3_ + MgO) = 20%, the conductivity reached 14.96 MS/m, and the hardness reached 100.23 HB. After heat treatment, the Cr phase was refined in the alloy, the conductivity increased from 14.96 MS/m to 18.27 MS/m, and the hardness increased from 100.23 HB to 103.1 HB.

## Figures and Tables

**Table 1:** Different CaO addition in raw materials (a—R = 10%, b—R = 15%, c—R = 20%, d—R = 25%).

Number	CuO	Cr_2_O_3_	Al	Al-40Mg	KClO_3_	CaO
a	1061.47	1737.55	695.92	99.43	239.68	165.94
b	1022.49	1673.75	683.30	97.63	264.03	258.79
c	978.80	1602.23	670.09	95.74	293.65	359.51
d	931.71	1525.14	655.59	93.66	324.91	468.97

**Table 2:** Content of substances in different reduction slags.

Number	Al_2_O_3_	Cr_2_O_3_	CaO	MgO
a	58.56	30.96	8.31	2.17
b	57.09	29.21	11.46	2.24
c	57.00	26.52	14.83	1.65
d	56.14	24.16	17.97	1.73

**Table 3:** Melting temperature of different reduction slags (°C).

Number	Initial Melting Temperature	Complete Melting Temperature
a	1666.94	2007.17
b	1659.44	1962.87
c	1649.05	1919.53
d	1546.80	1872.69

**Table 4:** Expressions of structural components and action concentration in CaO-Al_2_O_3_-Cr_2_O_3_-MgO slag system based on coexistence theory.

Number	Equation of Chemical Reaction	Mass Action Concentration
5	3(Ca^2+^+O^2−^) + Al_2_O_3_ = 3CaO·Al_2_O_3_	N5 = K_1_ × N_1_^3 × N_2_
6	(Ca^2+^+O^2−^) + 6Al_2_O_3_ = CaO·6Al_2_O_3_	N6 = K2 × N_1_ × N_2_^6
7	(Ca^2+^+O^2−^) + Al_2_O_3_ = CaO·Al_2_O_3_	N_7_ = K_3_ × N_1_ × N_2_
8	(Ca^2+^+O^2−^) + 2Al_2_O_3_ = CaO·2Al_2_O_3_	N_8_ = K_4_ × N_1_ × N_2_^2
9	(Ca^2+^+O^2−^) + (2Cr^3+^ + 3O^2−^) = CaO·Cr_2_O_3_	N_9_ = K_5_ × N_1_ × N_3_
10	(Mg^2+^ + O^2−^) + Al_2_O_3_ = MgO·Al_2_O_3_	N_10_ = K_6_ × N_4_ × N_2_
11	(Mg^2+^ + O^2−^) + (2Cr^3+^ + 3O^2−^) = MgO·Cr_2_O_3_	N_11_ = K_7_ × N_4_ × N_3_
12	(Ca^2+^ + O^2−^) + (Mg^2+^ + O^2−^) + Al_2_O_3_ = CaO·MgO·7Al_2_O_3_	N_12_ = K_8_ × N_1_ × N_2_ × N_4_
13	3(Ca^2+^ + O^2−^) + (Mg^2+^ + O^2−^) + 2Al_2_O_3_ = 3CaO·MgO·2Al_2_O_3_	N_13_ = K_9_ × N_1_^3 × N_2_^2 × N_4_
14	(Ca^2+^ + O^2−^) + 2(Mg^2+^ + O^2−^) + 8Al_2_O_3_ = CaO·2MgO·8Al_2_O_3_	N_14_ = K_10_ × N_1_ × N_2_^8 × N_4_^2

**Table 5:** Composition analysis of CuCr50 alloys synthesized in situ with different ingredients.

Alloy	Part	Cr	Cu	Al
a	Upper	46.53%	52.93%	0.33%
	Lower	46.74%	52.41%	0.35%
b	Upper	51.59%	47.41%	0.35%
	Lower	48.35%	50.77%	0.18%
c	Upper	47.09%	52.01%	0.30%
	Lower	46.61%	52.58%	0.39%
d	Upper	47.80%	51.65%	0.24%
	Lower	42.79%	56.43%	0.28%

**Table 6:** EDS results of CuCr alloys synthesized with different CaO ratios.

Number	Cu	Cr	Al	O	Mg
1	9.1	76.6	1.3	13	0
2	0.8	62.8	5.9	30.5	0
3	2.1	96.6	0.2	1.1	0
4	96.7	1.7	0.4	1.2	0.2
5	1.1	64.3	4.7	29.9	0
6	2.4	96.5	0.3	0.8	0
7	1.5	70.2	2.2	26.1	0
8	96.3	2.1	0.4	1.2	0
9	2.7	73.1	1.2	23	0
10	1.1	67.4	2.7	28.8	0
11	2.3	96.5	0.2	1	0.1
12	97.3	1.6	0.3	0.8	0
13	0.8	5.9	48.4	44.9	0
14	1.7	97.2	0.2	0.9	0
15	97.4	1.1	0.4	1	0.1

**Table 7:** Changes in grain boundary angle and properties of 20% CaO synthetic CuCr50 alloys.

Preparation Process	2–10°	10–60°	>60°	Conductivity MS/m	Hardness HB
As-cast	91.8%	8.21%	0.01%	14.96	100.23
Heat treatment	88.1%	11.9%	0.02%	18.27	103.10

## Data Availability

The original contributions presented in the study are included in the article, further inquiries can be directed to the corresponding author.
